# Altered gamma oscillations during pregnancy through loss of δ subunit-containing GABA_A_ receptors on parvalbumin interneurons

**DOI:** 10.3389/fncir.2013.00144

**Published:** 2013-09-17

**Authors:** Isabella Ferando, Istvan Mody

**Affiliations:** ^1^Departments of Neurology and Physiology, The David Geffen School of Medicine, University of CaliforniaLos Angeles, CA, USA; ^2^Interdepartmental Graduate Program in Molecular, Cellular, and Integrative Physiology, University of CaliforniaLos Angeles, CA, USA

**Keywords:** gamma oscillations, pregnancy, neurosteroids, GABA_A_ receptors, delta subunit, CA3 interneurons, parvalbumin, tonic inhibition

## Abstract

Gamma (γ) oscillations (30–120 Hz), an emergent property of neuronal networks, correlate with memory, cognition and encoding. In the hippocampal CA3 region, locally generated γ oscillations emerge through feedback between inhibitory parvalbumin-positive basket cells (PV+BCs) and the principal (pyramidal) cells. PV+BCs express δ-subunit-containing GABA_A_Rs (δ-GABA_A_Rs) and NMDA receptors (NMDA-Rs) that balance the frequency of γ oscillations. Neuroactive steroids (NS), such as the progesterone-derived (3α,5α)-3-hydroxy-pregnan-20-one (allopregnanolone; ALLO), modulate the expression of δ-GABA_A_Rs and the tonic conductance they mediate. Pregnancy produces large increases in ALLO and brain-region-specific homeostatic changes in δ-GABA_A_Rs expression. Here we show that in CA3, where most PV+ interneurons (INs) express δ-GABA_A_Rs, expression of δ-GABA_A_Rs on INs diminishes during pregnancy, but reverts to control levels within 48 h postpartum. These anatomical findings were corroborated by a pregnancy-related increase in the frequency of kainate-induced CA3 γ oscillations *in vitro* that could be countered by the NMDA-R antagonists D-AP5 and PPDA. Mimicking the typical hormonal conditions during pregnancy by supplementing 100 nM ALLO lowered the γ frequencies to levels found in virgin or postpartum mice. Our findings show that states of altered NS levels (e.g., pregnancy) may provoke perturbations in γ oscillatory activity through direct effects on the GABAergic system, and underscore the importance of δ-GABA_A_Rs homeostatic plasticity in maintaining constant network output despite large hormonal changes. Inaccurate coupling of NS levels to δ-GABA_A_R expression may facilitate abnormal neurological and psychiatric conditions such as epilepsy, post-partum depression, and post-partum psychosis, thus providing insights into potential new treatments.

## Introduction

Oscillations in cortical local field potentials in the γ-frequency band (30–120 Hz) reflect coordinated neuronal activity that manifests during different processing tasks such as memory and sensory encoding, and are considered important in adaptive functional organization of neuronal assemblies, spike-time dependent synaptic plasticity, and neurological performance (Singer, 1993; Paulsen and Moser, 1998; Sederberg et al., 2003; Montgomery and Buzsáki, 2007).

Frequency and power of γ oscillations result from a synchronized feedback dialogue between excitatory neurons and perisomatic inhibitory interneurons (INs). In particular, PV+BCs are the major contributors to the generation of γ oscillations, their activity is both necessary and sufficient to drive the rhythm, albeit other IN types may also play a regulatory role (Hájos et al., 2004; Mann et al., 2005; Cardin et al., 2009; Buzsáki and Wang, 2012). We have previously shown how γ oscillation frequency recorded in the CA3 *in vitro* is controlled by a δ-GABA_A_Rs-mediated tonic conductance of INs, that is dynamically balanced by an NMDA-R-mediated tonic excitation (Mann and Mody, 2010). Unlike its fast, synaptic GABA_A_Rs-mediated phasic counterpart, tonic inhibition is a slow persistent inhibitory conductance that is activated by ambient GABA, is mediated by extrasynaptic GABA_A_Rs, and decreases overall neuronal excitability by hyperpolarization or shunting inhibition (Brickley and Mody, 2012).

In the hippocampus, δ-GABA_A_Rs are predominantly expressed on dentate gyrus granule cells (DGGCs) and INs (Sperk et al., 1997). Regardless of cell specificity, all δ-GABA_A_Rs are uniquely sensitive to NS, including the progesterone-derived ALLO. NS are potent modulators of tonic inhibition, which they amplify by increasing GABA efficacy on δ-GABA_A_Rs (Stell et al., 2003; Meera et al., 2009). Moreover, NS can modulate network excitability by modifying δ-GABA_A_Rs surface expression (Maguire et al., 2005; Maguire and Mody, 2007), albeit candidate molecular mechanisms remain unknown. In mammals, brain ALLO follows oscillations in plasma progesterone, and during the last third of pregnancy, they both reach concentrations two orders of magnitude higher than any other physiological state (Paul and Purdy, 1992; Concas et al., 1998). These changes are paralleled by a downregulation of δ-GABA_A_Rs in DGGCs and CA1 pyramidal cells, in a compensatory homeostatic mechanism, which if altered, may lead to great imbalances in network excitability and postpartum behavior (Maguire and Mody, 2008; Maguire et al., 2009).

Thus far, δ-GABA_A_Rs plasticity has been reported only in neurons expressing α4-GABA_A_R (Smith et al., 1998; Sundström Poromaa et al., 2002; Maguire et al., 2005, 2009) that is considered natural partner of δ-GABA_A_R (McKernan and Whiting, 1996; Sur et al., 1999) in the forebrain. It is unclear whether INs, which usually express δ-GABA_A_Rs assembled with α1-GABA_A_Rs (Glykys et al., 2007) go through the same NS-related modifications. Moreover, as δ-GABA_A_Rs-mediated tonic conductance on PV+BCs controls γ oscillation frequency, it remains an open question whether γ oscillations are modulated during pregnancy. Deficits in PV+BCs output and consequent changes in γ oscillations have been reported in schizophrenia and may cause memory perturbations (Haenschel et al., 2009; Minzenberg et al., 2010). At the same time, the vast clinical evidence for pregnancy and postpartum-related psychiatric and neurological disturbances that vary from memory impairments to postpartum psychosis (Poser et al., 1986; Sit et al., 2006; Henry and Rendell, 2007) may also indicate a state of unrest in γ oscillations.

Our objective was to assess pregnancy-related δ-GABA_A_Rs plasticity in INs and possible changes in γ oscillations. We show that CA3 PV+INs express δ-GABA_A_Rs that become diminished during pregnancy. These anatomical findings are validated by a functional increase in kainate-induced γ oscillation frequencies driven by an IN-specific NMDA-R-dependent mechanism. Consistent with a very rapid plasticity process, pre-pregnancy δ-GABA_AR_ expression and γ oscillation frequencies are restored already at 48 h postpartum. The homeostatic nature of these alterations is demonstrated by our findings that physiological levels of ALLO found during pregnancy revert γ oscillations frequencies to control values.

## Materials and methods

### Animal handling

This study used adult (9–15 weeks of age) C57Bl/6 and mice lacking δ-GABA_A_Rs (*Gabrd*^−/−^ mice, on C57Bl/6 background) were housed with *ad libitum* access to food and water under the care of the UCLA Division of Laboratory Animal Medicine (DLAM). Mice were maintained on a light/dark cycle of 12 h, and all experiments were performed during the light period. Stress was minimized by moving the animals to the experimental area in their home cage at least 2 h prior to use during which time they were never handled. Virgin mice were anovulatory non-cycling females, pregnant mice were day-18 first time pregnant, postpartum mice were first time dams 48 h after parturition, and only if the pups were fed and cared for. Genotyping was performed by Transnetyx.

### Immunohistochemistry

Deeply anesthetized mice were transcardially perfused with 50 ml of 4% paraformaldehyde in 0.12 M phosphate buffer, at room temperature pH 7.3. Brains were postfixed overnight at 4°C, cryoprotected in 30% sucrose solution in Millonigs modified PBS, embedded in OCT compound (Andwin Scientific) on dry ice, and sectioned at −16°C with a cryostat (coronal, 35 μm). δ-GABA_A_Rs stains were processed under non-permeabilizing conditions in order to stain for membrane localized, functionally relevant receptors. Slices from virgin, pregnant and postpartum mice were processed in parallel. For diaminobenzidine (DAB) δ-GABA_A_Rs stain: endogenous peroxidases were quenched in methanol and 3% H_2_O_2_, 30 min. Slices were blocked in 10% normal goat serum (NGS), 2 h, incubated first with anti-δ-GABA_A_R antibody that recognizes the extracellular N-terminus of δ-GABA_A_R (1:500 in 10% NGS; generous gift from Dr. Werner Sieghart, Medizinische Universität, Wien, Austria) overnight, then with biotinylated goat anti-rabbit antibody (1:200 in 10% NGS; Vector Laboratories), 4 h. Signal was amplified with HRP-conjugated avidin enzyme complex (ABC Elite; Vector Laboratories), 30 min, then developed with DAB (Vector Lab). For DAB PV stain: 1.5%H_2_O_2_ in TBS, 10% normal horse serum (NHS) and 0.3% Triton X-100 as blocking agent, mouse anti-PV (1:5000 in 1:50 NHS; Swant), biotinylated horse anti-mouse (1:200 in 1:30 NHS; Vector Laboratories). For δ-GABA_A_Rs-PV double labeling: slices were incubated for 70 min at 90°C in a citrate buffer solution (0.05 M sodium citrate in 1% NaCl, pH 8.6) for antigen retrieval. Blocking was done in 10% NGS and 0.3% Triton X-100, 2 h. Slices were incubated in anti-δ-GABA_A_R and anti-PV (1:100; 1:5000 respectively, 0.1% NaN3 in TBS) for 4 days at room temperature, then in Cy3 and Cy5-conjugated goat anti-rabbit and anti-mouse antibodies, 2 h (1:750; Millipore). Slices were mounted on Superfrost Plus slides (Fisher Scientific), dehydrated (DAB stains only) and coverslipped with DPX Mountant for Histology (DAB stains, Sigma-Aldrich) or Fluoromount G (fluorescence stains, Southern Biotechnology).

### Microscopy and densitometric analysis

For bright field microscopy, digital images were taken with an Axioskop 2 Microscope and an AxioCam digital camera system and AxioVision 4.8 software (Zeiss). For the same magnification and the same staining images were taken under identical conditions of brightness and exposure time. The region of interest in CA3 was the whole CA3 stratum pyramidale (SP). The intensity of labeling was measured as optical density of the region of interest using NIH ImageJ software (Figure 4). As we did not find significant differences in background staining (which was minimal) between experimental groups, background subtraction was deemed unnecessary. For fluorescent microscopy, images were collected using a confocal microscope (Leica TCS-SP, Mannheim, Germany) equipped with Plan Fluor objectives connected to a camera (DP70, Olympus), and Leica confocal and DP70 camera software. Digital projection images of 35 μm *z*-stacks were assembled and analyzed using NIH ImageJ software. All images were captured under the same light intensity and exposure limits.

### Slice preparation and electrophysiology

Mice were anesthetized with halothane and decapitated following UCLA Chancellor's Animal Research Committee protocol. Horizontal 350 μm hippocampal slices were cut on a Leica VT1000S Vibratome in ice-cold N-Methyl-D-Glucamine (NMDG)-based HEPES-buffered cutting solution, containing in mM: NMDG 135, D-glucose 10, MgCl_2_ 4, CaCl_2_ 0.5, KCl 1, KH_2_PO_4_ 1.2, HEPES 26, Given the highly lipophilic nature 7.3–7.4, 295–297 mOsm/L, bubbled with 100% O_2_. Slices were incubated for 30 min at 32°C in an interface chamber in modified sucrose aCSF, containing in mM: NaCl 85, D-glucose 25, sucrose 55, KCl 2.5, NaH_2_PO_4_ 1.25, CaCl_2_ 0.5, MgCl_2_ 4, NaHCO_3_ 26, pH 7.3–7.4 when bubbled with 95% O_2_, 5% CO_2_. Modified aCSF was slowly substituted for normal aCSF at 32°C, containing in mM: NaCl 126, D-glucose 10, MgCl_2_ 2, CaCl_2_ 2, KCl 2.5, NaH_2_PO_4_ 1.25, Na Pyruvate 1.5, L-Glutamine 1, NaHCO_3_ 26, pH 7.3–7.4 when bubbled with 95% O_2_, 5% CO_2_. Recordings were done in an interface chamber at 35°C perfused with normal aCSF and 50 nM kainic acid (Tocris). Oscillatory network activity was recorded in CA3 SP with the use of a patch pipette filled with nACSF connected to a patch clamp amplifier (A-M Systems Inc., model 3000) sampled at 4096 Hz, band-pass filtered between 0.1 and 3,000 Hz, and an instrumentation amplifier (Brownlee BP Precision, model 210A). Field potentials were recorded using EVAN (custom-designed LabView-based software from Thotec) and analyzed with a custom written procedure (Wavemetrics, IGOR Pro 6.22A). Peak frequencies, power at peak frequency and total power were derived from the corresponding power spectral densities, calculated from 180 s period averages. Before and after drug treatment values are derived from the average power spectral density (psd) of 180 s periods before drug perfusion and 10 min after. D-AP5, PPDA, ALLO and finasteride were purchased from Tocris. ALLO and finasteride were dissolved in DMSO (final vehicle concentration 0.01%). Morlet wavelet transform was used to illustrate the power of γ-frequency band in time as previously described (Mann and Mody, 2010).

### Corticosterone measurements

Whole blood was collected at decapitation and plasma isolated by high-speed centrifugation. Plasma corticosterone levels were measured by enzyme-linked immunosorbent assay (Enzo Life Sciences). Absorbance was measured at 405 nm, and sample values derived from fitted standard binding curve. All samples and standards were run in parallel in the same plate.

### Data analysis

All data is shown as mean ± SEM. Statistical significance was determined at the 95% confidence interval with the use of statistical tests specified in each section.

## Results

### PV immunoreactivity remains unchanged in the CA3 at different gestational states

We first tested for gestational state-related anatomical alterations in PV+BCs. These INs display characteristic anatomical and firing properties and typically express the calcium-binding protein parvalbumin (PV), which is used as their anatomical hallmark (Klausberger et al., 2005, 2003; Freund and Katona, 2007). Although they are not the only hippocampal INs to express PV, they comprise the majority of PV+ INs (Baude et al., 2006), and together with cholecystokinin-expressing BCs (CCK+BCs) they are the only PV+INs to innervate the perisomatic region of principal cells (Freund and Katona, 2007; Klausberger and Somogyi, 2008). Until more cell-type specific protein markers are described, hippocampal SP PV immunolabeling is a good approximation to investigate PV+BCs anatomical distribution.

**Figure 1:**
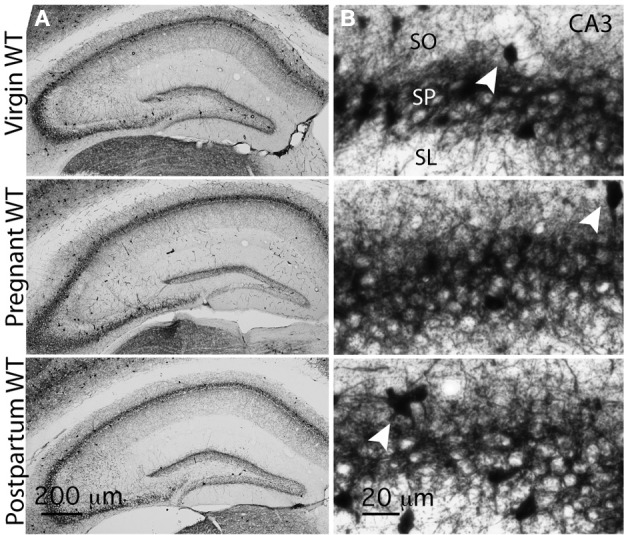
**PV distribution remains unchanged throughout the different gestational states. (A)** Representative bright-field images of whole hippocampal DAB staining for PV in virgin, pregnant and postpartum WT mice. PV+ terminals innervate CA1 and CA3 pyramidal cells and dentate gyrus granule cells, and form a plexus that wraps around their somata and proximal dendrites. **(B)** Representative high-magnification images of CA3 PV plexus in virgin, pregnant and postpartum WT mice. PV+IN somata are clearly visible within the stratum pyramidale (SP) and in its immediate vicinity (initial portion of stratum oriens SO and stratum lucidum SL), arrowheads. Optical density measurements in CA3 SP show no difference across gestational groups (in arbitrary units AU, mean ± SEM: virgin = 193.6 ± 0.9; pregnant = 192.9 ± 1.9; postpartum = 196.1 ± 1.7; *n* = 12, 10, 8 slices and *n* = 3 mice for each group.). One-Way ANOVA; *p* = 0.32, *F*_(2, 27)_ = 1.175.

Immunohistochemistry of immunoperoxidase staining for PV shows numerous PV immunopositive cell bodies and dense terminals surrounding the principal cells in hippocampal areas CA1, DG and CA3, consistent with previously reported distribution of PV staining in these structures (Figure 1A) (Gao and Fritschy, 1994; Freund and Buzsaki, 1996). CA3 PV immunoreactivity is preserved across the three experimental gestational groups. In order to assess potential changes specifically in PV+BCs innervation we carried out a densitometric analysis of PV staining in the SP, an area where most PV+ boutons belong to PV+BCs (Klausberger and Somogyi, 2008). No modification in CA3 PV plexus was detected through optical density (OD) measurements of CA3 SP (Figures 1B, 5).

The CA3 is an ideal brain region for the investigation of modulations in γ oscillations dependent on δ-GABA_A_Rs expression in INs. Here not only γ oscillations are locally generated, but also δ-GABA_A_Rs are exclusively expressed on INs as CA3 pyramidal cells tonic conductance is sustained solely by α5-GABA_A_Rs (Sperk et al., 1997; Glykys and Mody, 2006; Mann and Mody, 2010). In contrast, CA1 pyramidal cells and DGGCs use a combination of δ-GABA_A_Rs and α-GABA_A_Rs for their tonic conductance (Glykys et al., 2008), therefore making it hard to differentiate network effects of δ-GABA_A_Rs modulation on principal cells from that of INs.

### CA3 PV + INs express δ-GABA_A_Rs

CA3 γ oscillation frequency is controlled by a δ-GABA_A_Rs mediated tonic conductance of INs (Mann and Mody, 2010). PV+INs in the dentate gyrus have been shown to abundantly express δ-GABA_A_Rs (Yu et al., 2013). Alterations in these receptors during pregnancy could result in changes in γ frequency oscillations. Therefore, we sought to examine the presence of δ-GABA_A_Rs on PV+INs of the CA3 region, where kainate-dependent γ oscillations can be readily induced *in vitro*.

**Figure 2:**
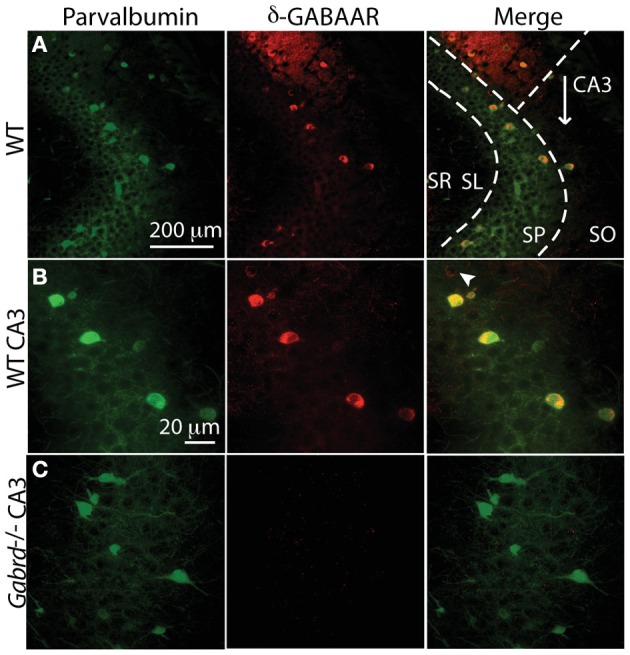
**The majority of CA3 PV+ interneurons also express δ-GABA_A_Rs. (A,B)** Immunohistochemical evidence of δ-GABA_A_Rs expression on CA3 PV interneurons in a WT mouse, by confocal microscopy. Green: PV, red: δ-GABA_A_Rs, yellow: colocalization. **(A**,**B)** PV and δ-GABA_A_Rs immunolabeling is strongest at the somata and it is clearly present and more faint in the processes around CA3 pyramidal cells. Most PV+ INs in the stratum pyramidale (SP) also co-localize δ-GABA_A_Rs; very rare interneurons are δ-GABA_A_Rs+ and PV+ (**B**, white arrowhead). Most CA3 PV+ and/or δ-GABA_A_Rs+ INs are concentrated around the SP, whereas they are sparser in stratum oriens (SO) stratum lucidum (SL) and stratum radiatum (SR). **(C)** Specificity of δ-GABA_A_Rs immunolabeling is confirmed by the lack of δ-GABA_A_R staining in a *Gabrd*^−/−^ mouse. No significant change in PV labeling is found in *Gabrd*^−/−^ mice.

Functional evidence of δ-GABA_A_Rs expression on most CA3 INs has been described (Mann and Mody, 2010), but a detailed anatomical study of δ-GABA_A_Rs expression in specific types of IN of the hippocampus has yet to be done. Here we demonstrate a large overlap of the two immunoreactivities in the somata of INs in area CA3 of the hippocampus (Figures 2A,B). Of 93 INs identified in 6 sections (35 μm thick) 3 sections apart in an area spanning across CA3-SP and 30 μm around it toward stratum oriens (SO) or stratum lucidum (SL), where most PV+BC somata are located, 85.2 ± 3.3% expressed both PV and δ-GABA_A_Rs, 11.7 ± 2.4% only PV and 3 ± 1.4% only δ-GABA_A_Rs. In the same slices, of the 31 labeled INs in the distal SO and stratum radiatum (SR), 38.9 ± 9.3% expressed both PV and δ-GABA_A_Rs, 31 ± 7.2% only PV and 30.1 ± 7.7% only δ-GABA_A_Rs. A χ^2^ analysis shows a highly significant difference between the two distributions. χ^2^ = 80.5, *p* < 0.0001, 2df. (Figure 2). Our peri-SP distribution is consistent with the findings of a recent work that showed almost complete overlap of PV and δ-GABA_A_Rs immunolabeling in DG INs proximal to the GC layer (Yu et al., 2013). Specificity of δ-GABA_A_Rs antisera was demonstrated with the use of a *Gabrd*^−/−^ male mouse as a negative control (Figure 2C). The importance of this control is particularly relevant for δ-GABA_A_Rs immunolabeling, as a commercially available δ-GABA_A_Rs specific antibody (Santa Cruz Biotechnology, SC-31438) showed unspecific binding in brain slices from *Gabrd*^−/−^ mice (Ferando et al., 2009).

Our results clearly show evidence for δ-GABA_A_Rs expression by CA3 PV+ INs. Additionally, as shown in Figure 2A, colabeling is also evident in hippocampal area CA1 INs and neuropil and the DG (data not shown).

### Surface δ-GABA_A_Rs expression decreases during pregnancy in INs of the pyramidal cell layer

**Figure 3:**
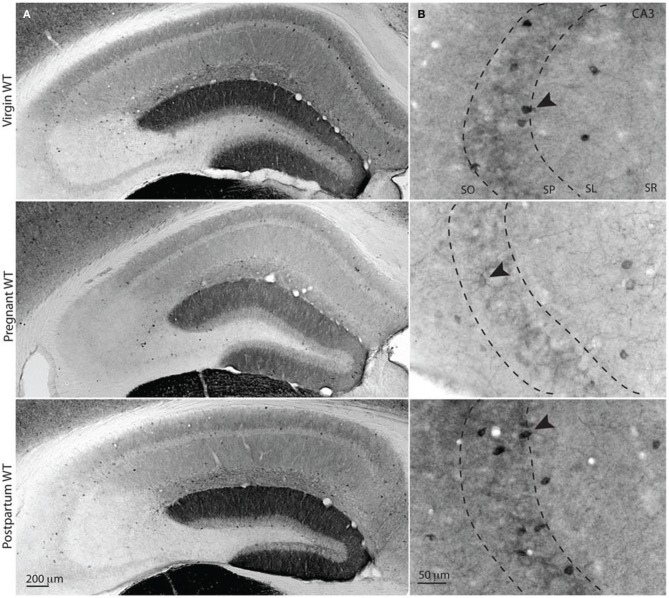
**Surface δ-GABA_A_Rs expression is reduced during pregnancy in CA3 stratum pyramidale interneurons. (A,B)** Representative bright-field images of whole hippocampal DAB staining for δ-GABA_A_Rs in virgin, pregnant and postpartum (48 h) WT mice. Surface staining of functionally relevant receptors was obtained by processing tissues under non-permeabilizing conditions. In the hippocampus δ-GABA_A_Rs are mostly expressed on dentate gyrus granule cell dendrites (forming dentate molecular layer), many hippocampal INs, and CA1 principal cells basal and apical dendrites. **(A)** Surface δ-GABA_A_Rs expression is reduced in hippocampal CA1 pyramidal cells and dentate gyrus granule cells, as previously reported, and this is reflected in a lighter staining of the neuropil in CA1 and DG. **(B)** δ-GABA_A_Rs expression in CA3 is exclusively found in interneuron somata, dendrites (black arrowheads) and in the interneuronal processes surrounding CA3 pyramidal cells (dotted lines show the boundaries of CA3 stratum pyramidale). In pregnant animals, CA3 δ-GABA_A_Rs surface expression is reduced, as suggested by lighter staining. Optical density measurements in CA3 SP are in AU mean ± SEM: virgin = 45.2 ± 2.4; pregnant = 15.7 ± 2.3; postpartum, 48.5 ± 1.7 CA3-SP total OD of bright-field images; *n* = 17, 10, 16 slices and *n* = 3 mice for each group. One-Way ANOVA followed by Tukey's multiple comparisons test; *p* < 0.0001 for virgin and pregnant and for pregnant and postpartum, and *p* > 0.05 for virgin and postpartum, *F*_(2, 40)_ = 58.95.

Pregnancy-related δ-GABA_A_Rs plasticity has been previously described in hippocampal principal neurons (Maguire et al., 2009). To assess whether modulation of δ-GABA_A_R expression in INs also plays a role in physiological and pathophysiological alterations during pregnancy and the postpartum, we stained slices of pregnant mice in parallel with slices of virgin and postpartum mice with δ-GABA_A_R-specific antisera (Figure 3). Pregnant mice were used for experiments at day-18 of pregnancy, in order to study long-term brain exposure to high levels of NS. Virgin mice were anovulatory, in order to avoid estrus cycle-linked modifications in δ-GABA_A_Rs previously described in the hippocampus (Maguire et al., 2005). Postpartum mice were used 48 h after parturition, when blood progesterone and ALLO levels become normalized to pre-pregnancy values (Concas et al., 1998).

Neuroactive steroid levels fluctuate with plasma progesterone and corticosterone levels. δ-GABA_A_R expression is modified at different time points in the ovarian cycle, and is influenced by short periods of acute stress (Maguire and Mody, 2007). In order to control for potential stress-related variations across animal groups, corticosterone plasma levels were measured. We found levels and variability similar to those previously reported for C57Bl/6 WT mice and *Gabrd*^−/−^ mice (Sarkar et al., 2011). No differences were found across groups (in ng/ml: virgin = 34.6 ± 16.3, pregnant = 41.6 ± 10.4, postpartum = 29.8 ± 16.5, *Gabrd*^−/−^ = 13.6 ± 3.2). One-Way ANOVA; *p* = 0.6159, *F*_(3, 27)_ = 0.6075.

In terms of δ-GABA_A_Rs anatomical distribution, while there is functional evidence for the existence of axonal δ-GABA_A_Rs on CA3 mossy fiber boutons (Trigo et al., 2008; Ruiz et al., 2010), we found δ-GABA_A_Rs immunoreactivity in hippocampal CA3 area to be restricted to the somata of INs and their terminal fields, which finely extend along the pyramidal cell layer as previously reported (Sperk et al., 1997; Peng et al., 2004).

Our findings show that most δ-GABA_A_Rs labeled interneuronal somata were localized in close proximity (within 30 μm) or within the CA3-SP. Interestingly, under non-permeabilizing conditions we find a significant decrease in the δ-GABA_A_R staining of hippocampal CA3 INs of pregnant mice, suggestive of a downregulation of functionally relevant δ-GABA_A_Rs on the surface of INs. Surface expression measured as optical density reverted to pre-pregnancy levels already 48 h postpartum (Figures 3, 5).

**Figure 4:**
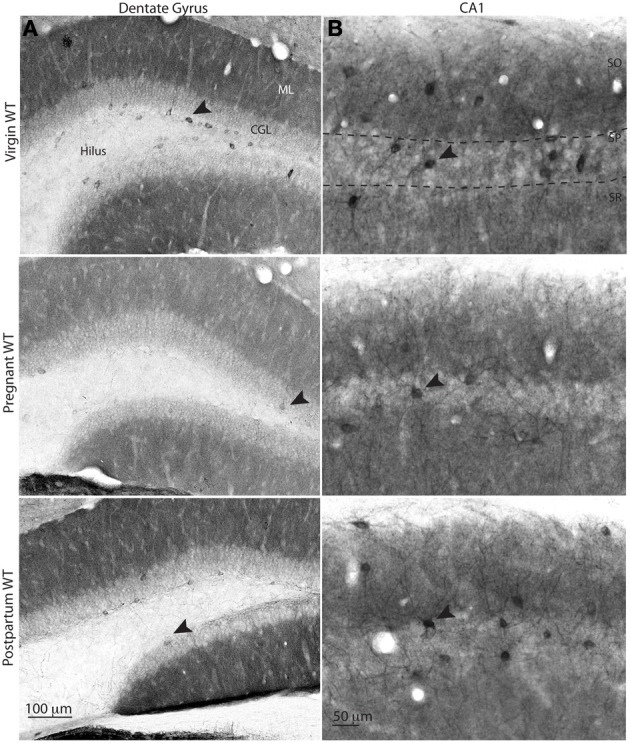
**δ-GABA_A_R immunolabeling of interneurons in the dentate gyrus and area CA1 of the hippocampus at different gestational states**. Representative bright-field images of δ-GABA_A_R staining in the dentate gyurs **(A)** and CA1 **(B)**. δ-GABA_A_R specific immunolabeling shows decreased staining of the INs that localize in the inner part of the granule cell layer and within CA1 stratum pyramidale (arrowheads). Some δ-GABA_A_R labeled INs are visible in the molecular layer of the dentate gyrus in virgin and postpartum animals, whereas no labeled INs are visible in slices from pregnant animals in this area.

We have previously described a functionally relevant decrease in δ-GABA_A_R immunostaining in hippocampal principal cells (DG molecular layer and CA1 SO and SR) during pregnancy (Maguire et al., 2009). Here we confirmed these modifications (Figure 4). Additionally, we demonstrate a brain-region specific upregulation to pre-pregnancy levels in the same areas during postpartum, consistent with previously described postpartum normalization of δ-GABA_A_Rs by use of whole hippocampal Western blot analysis (Maguire and Mody, 2008). Interestingly, similar to our findings in the CA3 region, the staining of INs in the CA1 and DG regions is also suggestive of surface δ-GABA_A_Rs downregulation during pregnancy, as δ-GABA_A_R-labeled INs consistently appear less numerous and less strongly immunoreactive (Figure 4). Moreover, just like in the CA3 region, downregulation of δ-GABA_A_Rs on INs reverted to pre-pregnancy levels in the immediate postpartum. In the DG, labeled INs were mostly localized around the inner granule cell layer, and some sparse INs could be found in the molecular layer as previously described (Peng et al., 2004; Glykys et al., 2007) (Figure 4). Similar findings are also evident in hippocampal area CA1, where most affected INs appear those in the immediate proximity of the pyramidal cell layer.

**Figure 5:**
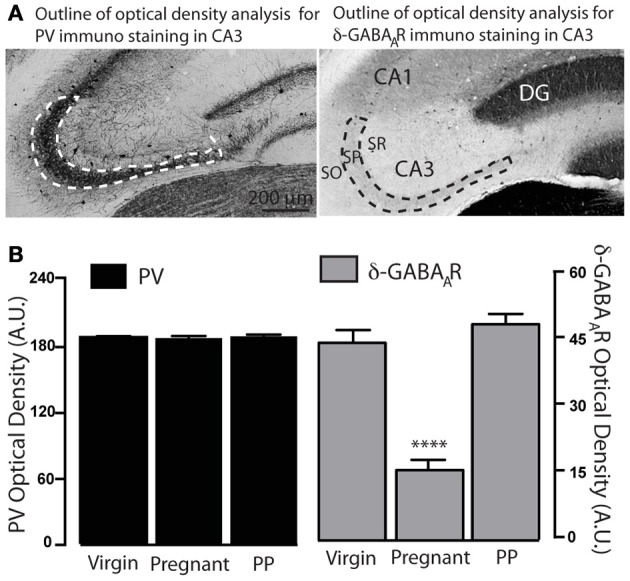
**Semi quantitative optical density analysis of PV and δ-GABA_A_R immunolabeling across different gestational states. (A)** Representative bright-field images of CA3 PV and δ-GABA_A_R-specific DAB staining. Dotted lines delimit the boundaries of the area analyzed (CA3 stratum pyramidale) for optical density measurements. **(B)** Optical density measurements (arbitrary units, A.U.) show a significant reduction in δ-GABA_A_R expression in pregnant animals. No difference is found in PV expression across gestational states. Asterisks denote significance, *p* < 0.0001.

In CA3, CA1 and DG anatomical localization of those INs in which δ-GABA_A_R expression is mostly affected during pregnancy is suggestive of BCs, as cell bodies of this class of INs are normally found in close proximity of the principal cell layer, in an ideal position to make preferential contacts with their perisomatic region (Freund and Buzsaki, 1996; Klausberger and Somogyi, 2008).

### *In vitro* CA3 γ oscillation frequency is increased during pregnancy

In the light of our anatomical findings we decided to examine a PV+BCs dependent network behavior, γ oscillations, as their frequency is controlled by δ-GABA_A_R-mediated tonic inhibition of INs. As a result, *in vitro* experiments on *Gabrd*^−/−^ mice show a constitutively higher frequency both in cholinergically-induced and kainate-induced γ oscillations in the CA3, compared to WT mice (Mann and Mody, 2010). Given these previous findings, we addressed the question whether δ-GABA_A_R plasticity on CA3 INs of pregnant mice has functional consequences on CA3 γ oscillations. Oscillations are defined by their frequency and power and result from the periodically timed feedback interaction between INs and principal neurons (Mann et al., 2005).

**Figure 6:**
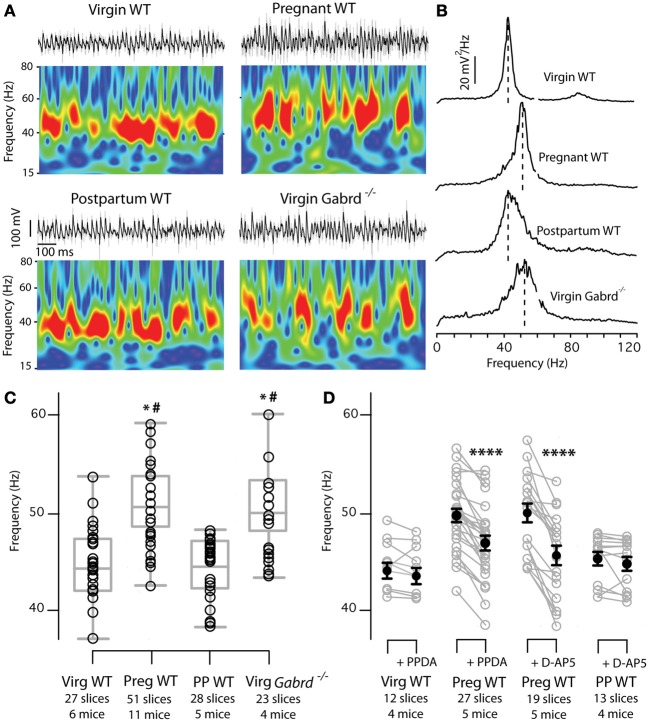
**γ Oscillations frequency is increased in the CA3 of pregnant animals in the absence of physiological ALLO levels**. Increased CA3 γ oscillations frequency in slices of WT pregnant mice is sustained by NMDA-Rs activation on interneurons. **(A)** Kainate induced γ oscillations (50 nM) recorded extracellularly in CA3 stratum pyramidale at different gestational states show higher frequency in slices of WT pregnant and *Gabrd*^−/−^ virgin mice compared to slices from WT virgin and postpartum mice. Upper traces: representative 1 s epochs of LFPs band-pass filtered between 15 and 120 Hz (black) and raw traces (gray). Morlet wavelet transforms of the corresponding traces show γ oscillatory behavior. Warmer colors represent higher power, and the same scale has been used for all four wavelets. **(B)** Plots of power spectral density calculated over 180 s periods of the same recordings as in **(A)**. Note increased γ frequency in slices from pregnant and *Gabrd*^−/−^ mice. No differences are found in power at peak frequency or total power (30–120 Hz) **(C)**, Box plots showing peak frequencies for the four experimental groups. Each dot symbolizes the peak frequency of one slice calculated as the frequency with the highest power in a 180 s period power spectral density as in **(B)**. Box plots represent mean, 25th and 75th percentile, and largest and smallest values. Significance established by One-Way ANOVA followed by Turkey's multiple comparisons test. ^*^p < 0.0001 between WT pregnant and WT virgin or WT postpartum respectively. ^#^*p* < 0.0001 between *Gabrd*^−/−^ virgin and WT virgin or WT postpartum respectively. **(D)** In slices of WT pregnant mice bath application of the NMDA-R subunit-unspecific antagonists D-AP5 (25 μ M) or GluN2D-containing NMDA-Rs specific antagonist PPDA (1μ M) decreases γ oscillations frequency to WT virgin and WT postpartum (PP) values. The same drugs have no effect on γ frequencies of slices of WT virgin or WT postpartum mice. Mean frequency ± SEM in Hz, significance established by two-tailed paired *t*-test: pregnant PPDA = 49.7 ± 0.7 to 46.9 ± 0.7, *p* < 0.0001, *n* = 27 slices, 5 mice; virgin PPDA = 44.0 ± 0.8 to 43.5 ± 0.9 *p* = 0.1, *n* = 12 slices, 4 mice; pregnant D-AP5 = 49.8 ± 0.96 to 45.6 ± 1.0 *p* < 0.0001, *n* = 19 slices, 5 mice; postpartum D-AP5 = 45. 5 ± 0.68 to 45.1 ± 0.68 *p* = 0.2, *n* = 13 slices, 4 mice. Asterisks denote significance. *n*'s for each group are reported in the figure.

We found a statistically significant increase in the peak frequency of kainate-induced γ oscillations in slices obtained from pregnant mice (Figures 6A–C). This resembled the increased frequency found in *Gabrd*^−/−^ virgin mice. γ oscillations in slices from virgin and postpartum WT mice have similar, lower frequencies (Figures 6A–C). We found no differences across groups in power at peak frequency or in total power (between 30 and 120 Hz) (Table 1). These findings are consistent with the previously reported similar power of γ oscillations of WT and *Gabrd*^−/−^ males (Mann and Mody, 2010). Statistical significance was determined by One-Way ANOVA followed by Tukey's multiple comparisons test for peak frequency: *p* < 0.0001, *F*_(3, 125)_ = 28.12; for power at peak frequency: *p* = 0.32, *F*_(3, 125)_ = 1.168; for total power (30–120 Hz): *p* = 0.27, *F*_(3, 125)_ = 1.338.

**Table 1:** **Details of γ oscillations characteristics by gestational state and genotype**.

	**Virgin WT**		**Virgin ***Gabrd***^−/−^**	**Pregnant WT**					**Postpartum WT**	
	**Control**	**+PPDA (1 μM)**	**Control**	**Control**	**+D-AP5 (25 μM)**	**+PPDA (1 μM)**	**+DMSO**	**+ALLO (100 nM)**	**Control**	**+D-AP5 (25 μM)**
Frequency (Hz)	44.9 ± 0.7	43.5 ± 0.8	50.5 ± 0.8^*^	50.8 ± 0.5^*^	45.6 ± 1^*^	46.9 ± 0.7^*^	50 ± 1^*^	45.2 ± 1^*^	44.6 ± 0.6	45.1 ± 0.7
Power at peak frequency (E-11 V^2^s^−1^)	4.87 ± 1.05	3.96 ± 1.24	4.6 ± 0.93	7.22 ± 1.3	7.53 ± 1.38	5.19 ± 1.49	6.34 ± 1.33	10.2 ± 5.94	5.05 ± 0.99	4.52 ± 1.31
Total power 30–120 s^−1^ (E-10 V^2^)	8.54 ± 1.32	8.23 ± 1.94	10.7 ± 1.76	14.1 ± 2.25	14.3 ± 2.35	11.5 ± 2.79	13.7 ± 3.02	12.1E ± 2.97	11.4 ± 1.9	10.8 ± 3
*n* slices (mice)	27 (6)	12 (4)	23 (4)	51 (11)	19 (5)	27 (5)	21 (5)	14 (5)	28 (5)	13 (3)

Summary of mean values and SEM for each of the gestational states and genotypes derived from the corresponding 180 s period averages power spectral densities. γ oscillations were characterized by their peak frequency, power at peak frequency and total power (30–120 Hz). Asterisks denote significance calculated by One-Way ANOVA followed by Turkey's multiple comparisons test (for controls), or two-tailed paired t-test (for DAP-5 and PPDA treatment), or two-tailed unpaired t-test (for DMSO and ALLO incubation).

For each gestational state we tested for individual variability or slice location differences (septal through temporal) in peak frequency, power at peak frequency and total power by One-Way ANOVA, and found no significant differences. All *p*-values > 0.05, F(DFn, DFd) for peak frequency, power at peak frequency and total power (30–120 Hz) as follows: virgin WT, individual variability *F*_(5, 21)_ = 1.267, 1.136, 1.966; slice location *F*_(5, 21)_ = 0.418, 0.69, 0.9. Pregnant WT, individual variability *F*_(10, 40)_ = 1.86, 0.66, 1.27; slice location *F*_(6, 44)_ = 1.13, 1.27, 1.73. Postpartum WT, individual variability *F*_(4, 23)_ = 1.5, 0.66, 0.65; slice location *F*_(6, 21)_ = 1.56, 0.72, 0.66. Virgin *Gabrd*^−/−^, individual variability *F*_(3, 19)_ = 0.28, 1.38, 1.68; slice location, *F*_(5, 17)_ = 0.18, 1.16, 0.98. These findings are consistent with the idea that kainate-induced γ oscillations *in vitro* are homogeneous throughout the septo-temporal axis of the hippocampus and vary little across different animals.

Our electrophysiological results corroborate the anatomical finding of decreased CA3 interneuronal δ-GABA_A_R expression in pregnant mice with an increase in the frequency of kainate-induced γ oscillations during pregnancy. Interestingly, γ power remained unchanged across the experimental groups, suggesting that only γ oscillations frequency is under the control of interneuronal δ-GABA_A_Rs. Additionally, we showed that γ band oscillations are increased in frequency in *Gabrd*^−/−^ females as it was previously demonstrated in *Gabrd*^−/−^ males (Mann and Mody, 2010).

### Increased γ oscillation frequency results from imbalance between activation of NMDA-Rs and δ-GABA_A_Rs on INs

The switch of γ oscillations to higher frequencies in the face of reduced δ-GABA_A_Rs on CA3 INs most likely results from a reduced tonic GABA conductance in these cells, which translates into an enhanced NMDA-R-mediated tonic excitation (Mann and Mody, 2010). This is thought to be a control mechanism that allows for ample modulatory ability and a large dynamic range of the γ oscillations *in vivo*. Although at near physiological temperature (35°C) *in vitro* CA3 γ oscillations are quite stereotyped, there is large variability of γ frequencies *in vivo* within the same animal (Colgin et al., 2009). An equilibrium between tonic inhibition and excitation on the neurons that generate and maintain the oscillations may be a mechanism by which γ frequencies are modulated (Mann and Mody, 2010).

In order to determine if a similar balancing mechanism is also responsible for the increased γ frequency found in slices from pregnant mice, we tested the effect on γ frequencies of two NMDA-R antagonists: the wide spectrum antagonist 2-amino-5-phosphonopentanoic acid (D-AP5), and a drug (2S^*^,3R^*^)-1-(phenanthren-2-carbonyl)piperazine-2,3-dicarboxylic acid (PPDA) that at low concentrations preferentially antagonizes GluN2D-containing NMDA-Rs (Feng et al., 2004; Morley et al., 2005; Lozovaya et al., 2004). As interneuronal NMDA-Rs are particularly enriched in GluN2D subunits (Monyer et al., 1994; Standaert et al., 1996), the use of the latter compound addresses whether the increased γ oscillation frequency results from the activation of NMDA-Rs on INs. D-AP5 (25 μ M) significantly reduced γ frequency in slices of pregnant mice to levels comparable to pre-pregnancy values (Figure 6D; Table 1). At the same time, D-AP5 was ineffective in modifying γ frequency in slices of postpartum mice.

Consistent with the D-AP5 findings, PPDA (1 μ M) decreased γ oscillation frequency in slices from pregnant mice (Figure 6D; Table 1) and was ineffective in modifying γ oscillation frequency in slices from virgin mice. D-AP5 and PPDA had no effects on power at peak frequency and total power (between 30 and 120 Hz), *p* > 0.05 by two-tailed paired *t*-test (Table 1). One-Way ANOVA comparison of the 4 frequency groups after NMDA-R block (pregnant after D-AP5, pregnant after PPDA, virgin after PPDA, postpartum after DAP-5) shows no difference across groups, *p* = 0.16; *F*_(3, 99)_ = 1.752.

These data demonstrate that the increased γ oscillation frequency observed in slices of pregnant mice is sustained by a PV+INs specific mechanism involving enhanced NMDA-R-mediated tonic excitation following the reduction of the δ-GABA_A_Rs-mediated tonic conductance. This appears to be the result of the pregnancy-related downregulation of δ-GABA_A_Rs in INs. The increase in γ frequency is thus mediated by a mechanism similar to that described for *Gabrd*^−/−^ mice (Mann and Mody, 2010). The virgin and postpartum experimental groups were not sensitive to NMDA-R blockade, consistent with the idea that the activation of δ-GABA_A_Rs on INs is sufficient to keep the tonic excitation in check.

### Exposure to ALLO levels found in pregnancy (100 nM) lowers the frequency of γ oscillations to pre-pregnancy values

**Figure 7:**
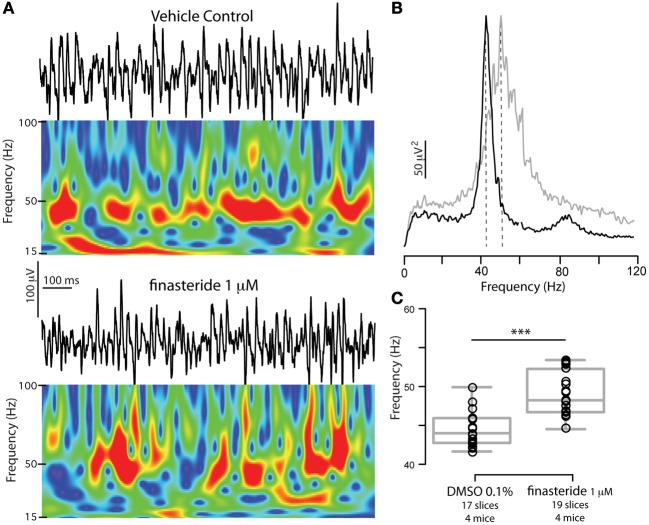
**Blocking neurosteroidogenesis increases γ oscillations frequency in slices from WT mice. (A)** Representative 1 s epoch of local field potential oscillations and corresponding Morlet Wavelets in the CA3 region of hippocampal slices obtained from a WT adult male mouse in vehicle (DMSO 0.01%) or in 1 μ M finasteride (after 30 min incubation). **(B)** Power spectral densities of the same recordings (average of 180 s) for DMSO 0.01% (black) and finasteride (gray). **(C)** Peak frequencies of γ oscillations recorded in either vehicle or 1 μ M finasteride. The latter have significantly higher peak frequencies. Box plots represent mean, 25th and 75th percentile, and largest and smallest values. Mean peak frequency ± SEM in Hz: DMSO 0.01% = 44.5 ± 0.5, finasteride = 49.1 ± 0.6 *p* < 0.0001, two-tailed unpaired *t*-test. Asterisks denote significance (*p* < 0.05). No differences were found in power at peak frequency, *p* = 0.5, and total power (30–120 Hz), *p* = 1. *n*'s for each group are reported in the figure.

Given the highly lipophilic nature of NS, it was suggested that they may access their binding sites on GABA_A_Rs after accumulation and lateral diffusion in the plasma membrane (Chisari et al., 2010). Whether during *in vitro* preparations of brain slices NS dissolved in plasma membranes are completely washed off remains to be fully established although some evidence would suggest at least partial depletion. Several *in vitro* experiments using finasteride, an inhibitor of 5α-reductase (a key enzyme in the local NS synthesis pathway), have unveiled the existence of NS synthesis in slices (Belelli and Lambert, 2005). This suggests that NS synthetized prior to the enzymatic block are either degraded or washed off during *in vitro* incubation. To confirm this depletion in our slices, in a separate set of experiments in slices from WT males we noticed a significant increase in γ oscillation frequency after 30 min of incubation in 1 μ M finasteride compared to slices incubated in vehicle alone (Figure 7). As δ-GABA_A_Rs respond poorly to GABA in the absence of NS, this finding is consistent with the wash-out of NS from slices, following pharmacological blockade of local NS synthesis. NS presence in slices likely result from continuous enzymatic conversion of local precursors, namely steroids synthetized *de novo* from cholesterol (Rupprecht et al., 2010), rather than the *in vivo* NS still bound to the plasma membrane after slice preparation. Since during pregnancy most of brain ALLO is derived from plasma progesterone (Paul and Purdy, 1992; Concas et al., 1998), it is reasonable to assume that slices incubated in a progesterone- and ALLO-free nACSF will be devoid of the NS levels found in the brains of pregnant mice. Consequently, *in vitro* brain preparations of pregnant mice will suffer an acute withdrawal of NS from plasma precursors, making synthesis from local precursors the only enzymatic pathway for maintaining NS levels. In support of this idea, we previously published evidence of altered slice excitability in slices from pregnant mice in the absence of physiological pregnancy levels of ALLO (Maguire et al., 2009).

**Figure 8:**
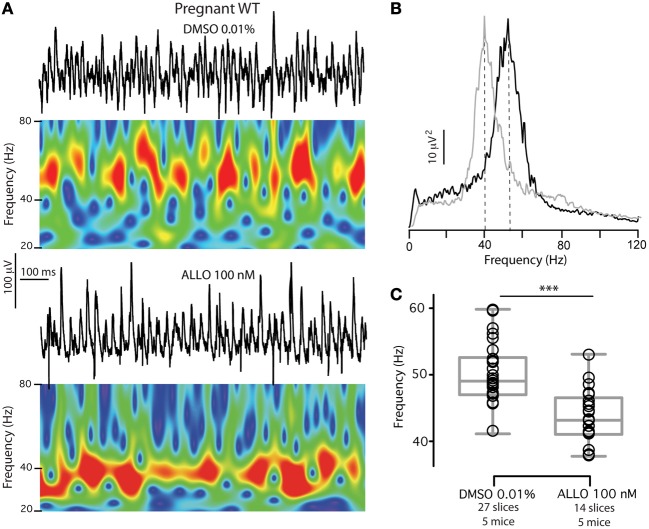
**Exposure to ALLO levels found in pregnancy (100 nM) reverts CA3 γ oscillations frequency in pregnant mice to control values. (A)** Representative 1 s epoch of local field potential oscillations and corresponding Morlet wavelet transforms of 1 s epochs of local field potentials recorded in the presence of 50 nM kainate depict γ oscillatory behavior over time. Slices from the same pregnant WT animal were incubated and recorded in either vehicle (DMSO 0.01%) or ALLO 100 nM. Slices incubated in vehicle show significantly higher γ oscillations frequency compared to slices incubated in ALLO. **(B)** Power spectral densities of the same recordings (average of 180 s) for DMSO 0.01% (black) and ALLO (gray). **(C)** Box plots summarizing peak frequencies of the two experimental groups and representing mean, 25th and 75th percentile, and largest and smallest values. *n*'s for each group are reported in the figure. Significance established by two-tailed unpaired *t*-test, *p* < 0.0001.

Therefore, in order to determine whether interneuronal δ-GABA_A_Rs downregulation and the correlated increase in γ frequency during pregnancy could be ascribed to a homeostatic mechanism which counterbalanced increased NS levels with δ-GABA_A_Rs downregulation, we tested the effects of physiological pregnancy levels of ALLO (100 nM) (Paul and Purdy, 1992; Concas et al., 1998) on γ oscillations frequency. Slices of pregnant mice were incubated from the time of cutting to the time of recording in either vehicle (0.01% DMSO) or 100 nM ALLO. Slices from the same animal were randomly assigned to either one experimental group and oscillations were recorded. We found that slices incubated in DMSO had γ oscillation peak frequencies similar to those recorded in slices of pregnant mice not exposed to the vehicle (two-tailed unpaired *t*-test, *p* = 0.2). Moreover, 100 nM ALLO was capable of significantly lowering peak frequency to values comparable to virgin and postpartum slices, *p* < 0.0001 by two-tailed unpaired *t*-test (Table 1; Figure 8). Exposure to ALLO did not affect power at peak frequency or total power (between 30 and 120 Hz). These findings demonstrate that under experimental conditions similar to physiological states during pregnancy, network output remains constant. In particular, γ oscillation frequencies are regulated by the levels of brain NS and the amount of δ-GABA_A_Rs expressed on INs. The inability to appropriately and timely regulate δ-GABA_A_Rs expression on INs or NS synthesis may predispose to or facilitate states of altered network oscillatory activity.

## Discussion

This study demonstrates a homeostatic δ-GABA_A_R plasticity in mouse hippocampal INs during pregnancy and postpartum. Immunohistochemical findings showed a transient δ-GABA_A_Rs downregulation in INs during the last third of pregnancy, which was fully reversible in the early postpartum. This led to altered network dynamics after the acute *in vitro* withdrawal of the high levels of ALLO found in pregnancy manifested in increased γ oscillation frequency in the hippocampal CA3 region of pregnant mice. This increase was fully reversible either by blocking interneuron-specific NMDA-Rs, or by restoring ALLO levels in the slices. Our findings are consistent with the idea that a δ-GABA_A_R-mediated tonic conductance of CA3 INs controls γ oscillation frequency by modulation of NMDA-R-mediated tonic excitation (Mann and Mody, 2010).

The observation that gamma oscillation frequency in slices from mice with partially downregulated δ-GABA_A_R expression closely resembles gamma oscillation frequencies found in slices obtained from *Gabrd*^−/−^ mice may not be unexpected (see similar dentate excitability between *Gabrd*^−/−^ and pregnant WT in the absence of ALLO, in Maguire et al., 2009). It is possible that in the total absence of δ-GABA_A_Rs, other tonically active GABA_A_Rs may be upregulated, but this hypothesis will require further investigation. Our findings in the present paper about the effects of partial δ-GABA_A_Rs reduction in PV+ INs during pregnancy were validated in mice heterozygous for δ-GABA_A_R expression only in PV+ cells (data not shown). In these mice, as in pregnant mice, *in vitro* γ oscillations are significantly faster compared to WT mice. Thus, it is possible that a partial reduction δ-GABA_A_Rs in PV+INs results in a full activation of NMDA-Rs in the same cells that can be no longer enhanced by further deletion of δ-GABA(A)Rs.

In the CNS δ-GABA_A_Rs are found on both principal cells and INs. In neocortical INs, δ-GABA_A_Rs are thought to be mostly expressed in neurogliaform cells (Oláh et al., 2009). Nevertheless, the modulation of γ oscillation frequency by a THIP-sensitive (synthetic δ-GABA_A_R-specific agonist) tonic current of CA3 INs (Mann and Mody, 2010), suggests that in the hippocampus δ-GABA_A_Rs expression is also present in other types of IN, at least in PV+BCs, the IN type mainly responsible for generation of γ oscillations (Buzsáki and Wang, 2012). The anatomical confinement of δ-GABA_A_Rs to PV+INs, evidenced by the very low ratio (3%) of PV+negative δ-GABA_A_Rs expressing INs around the SP, also suggests that CCK+BCs do not express δ-GABA_A_Rs. If confirmed by more detailed future studies, this finding could open interesting functional implications particularly since hippocampal CCK+BCs do not seem to express α 1-GABA_A_Rs (Gao and Fritschy, 1994), a natural partner of δ-GABA_A_Rs in INs (Glykys et al., 2007). Endogenous and exogenous modulators of δ-GABA_A_R-mediated tonic conductance, such as NS and EtOH, by influencing only PV+BCs through δ-GABA_A_Rs, will dynamically shift the weight between the two types of perisomatic inhibition (Freund and Katona, 2007). It is remarkable that PV+BCs, defined as the “orderly clockwork” of the hippocampus compared to the “variable fine-tuning” role of CCK+BCs (Freund and Katona, 2007), would preferentially express δ-GABA_A_Rs sensitizing them to constantly changing molecules (e.g., NS). If PV+BCs use NS for instantaneous modifications of network dynamics in response to behavioral needs, the compensatory downregulation in δ-GABA_A_Rs expression during pregnancy suggest that network oscillatory activity is indeed a functional neuronal output that is kept under strict control in terms of consistency, reliability, and “order.” PV+BCs are not the only PV+expressing INs in CA3, although (at least in CA1) they are the majority (Baude et al., 2006). Axoaxonic cells and bistratified cells are other PV+INs, but these INs don't participate much in γ oscillations (Gulyás et al., 2010; Dugladze et al., 2012). Therefore, they are unlikely to be involved in changes in γ activity following δ-GABA_A_Rs modulation. Moreover, these latter PV+INs have been shown to express substantially less extrasynaptic α 1-GABA_A_Rs and consequently have lower levels of tonic inhibition (Baude et al., 2006; Gao and Fritschy, 1994).

The relationship between δ-GABA_A_R plasticity and its partnership with various α subunits is an important issue. We have previously shown how swings in brain NS content will affect network output not only by increasing neuronal tonic conductance, but also by regulating surface δ-GABA_A_Rs expression (Maguire et al., 2005; Maguire and Mody, 2007). Similar modulations in α 4-GABA_A_Rs, the specific partner of δ-GABA_A_Rs in principal neurons, have also been observed (Smith et al., 1998; Follesa et al., 2001). In addition, studies in *Gabra*4^−/−^ mice showed how surface δ-GABA_A_Rs expression in excitatory neurons depends on the presence of α4-GABA_A_Rs (Glykys et al., 2007; Chandra et al., 2006). These findings together with the established plastic nature of α4-GABA_A_Rs (Roberts et al., 2005, 2006) led to the notion that perhaps the δ-GABA_A_R plasticity observed during altered NS levels depends on α4-GABA_A_Rs (Shen et al., 2007, 2010; Kuver et al., 2012). However, in INs δ-GABA_A_Rs naturally pair-up with α 1-GABA_A_Rs (Glykys et al., 2007) and although δ/α1-GABA_A_Rs are as sensitive to NS as δ/α4-GABA_A_Rs (Bianchi and Macdonald, 2003; Meera et al., 2009) the question whether δ/α 1-GABA_A_R expression is also regulated following NS oscillations remained open. Ours is the first report of δ-GABA_A_R plasticity in neurons with no α4-GABA_A_Rs. Although δ-GABA_A_R upregulation in molecular layer INs of the DG has been proposed in a mouse model of epilepsy, a concurrent reduction in DG neuropil labeling made quantification somewhat difficult (Peng et al., 2004).

Acute exposure to levels of NS found in pregnancy leads to sedation and anesthesia (Child et al., 1971; Carl et al., 1990; Rupprecht, 2003), hence during pregnancy the mammalian brain faces the challenge to maintain an overall functional network, despite large hormonal changes. Indeed many women experience mild to severe disturbances in their neurological performance, mostly during times of fast rise or drop in progesterone and its neuroactive metabolites (Poser et al., 1986). We have previously proposed a model for δ-GABA_A_Rs downregulation in excitatory neurons as a homeostatic mechanism of adaptation that allows these cells to maintain a constant level of excitability throughout pregnancy (Maguire et al., 2009).

δ-GABA_A_Rs plasticity on DGGC or DG INs can be ruled out as possible player in the observed increased frequency of CA3 γ oscillations. In fact, Although DG can sustain gamma oscillatory activity which can couple with that of CA3 (Akam et al., 2012), *in vivo* and *in vitro* studies have shown how the DG doesn't host an endogenous oscillator, on the contrary it needs intact afferent entorhinal connections in order to oscillate at γ frequency (Bragin et al., 1995; Csicsvari et al., 2003). In addition, in (Mann and Mody, 2010), some experiments were done after isolating CA3 from the DG without any apparent effects on the characteristics of γ oscillations in CA3. Lastly, in the presence of 50 nM kainate the LFP activity in the DG is unaffected. For these reasons we propose the observed shift in frequency depends solely on δ-GABA_A_Rs modulation on CA3 interneurons.

Here we describe a pregnancy-dependent loss of δ-GABA_A_Rs in CA3-SP INs, which was reversible within 48 h postpartum, and seems to be homeostatic in nature. In addition to the δ-GABA_A_R downregulation in CA3-SP INs, we show similar changes in CA1-SP and DG-INs. Numerous cortical INs also express δ-GABA_A_Rs, and it is likely that δ-GABA_A_Rs plasticity during pregnancy occurs in some or all of these cells. Identification of the specific types of INs modifying δ-GABA_A_Rs expression during pregnancy or other periods of steroid hormone changes (ovarian cycle or stress), and the functional consequences of this plasticity will require further studies. Although the IN-δ-GABA_A_R plasticity during pregnancy and the postpartum period likely affects different IN types, the increase in γ oscillations frequency is the functional consequence of pregnancy-related δ-GABA_A_Rs loss specific to PV+BCs. The expression of PV in BCs decreases in patients with schizophrenia (Lewis et al., 2005, 2012), and this is correlated with modifications in γ oscillation activity, although the exact underlying mechanism remains to be established (Uhlhaas and Singer, 2010, 2012). The changes in γ oscillatory activity during pregnancy in our study did not result from changes in PV immunoreactivity across different gestational states, but from the plasticity of a specific GABA_A_R subunit on these INs. Other, still to be uncovered, molecular and cellular modifications in PV+INs may also contribute to altered γ oscillatory activity and may lead to convergent psychiatric syndromes.

Slices prepared from pregnant mice are subject to an artificial acute NS withdrawal as plasma-derived precursors and NS are washed out during nASCF perfusion. Addition of 100 nM ALLO to nACSF completely restored γ oscillation frequencies to virgin and postpartum values consistent with the homeostatic nature of IN-δ-GABA_A_Rs downregulation during pregnancy and with the dependency of γ oscillations frequency on a NS-regulated δ-GABA_A_Rs system. Modifications in network activity are only revealed after abrupt *in vitro* ALLO withdrawal indicating the natural propensity of the network to adapt to large hormonal swings. However, this inherent plasticity may expose the brain to ineffective network oscillatory dynamics in the case of exaggerated or untimely NS modifications, or to inadequate adjustment of δ-GABA_A_Rs expression. Moreover, the physiological control of network dynamics exerted by NS fluctuations could be potentially less adaptable during pregnancy. A recent report showed concentration-dependent dual effects of GABA on the inhibitory or excitatory nature of IN tonic conductance (Song et al., 2011). In our study we did not perform direct tonic GABA conductance recordings from PV+BCs, but the complete reversibility of γ oscillation frequency to lower values after NMDA-R blockade in INs when IN δ-GABA_A_Rs are diminished is consistent with an inhibitory role of the tonic GABA conductance in PV+BCs. During pregnancy, CA3 PV+BCs must have a decreased inhibitory (hyperpolarizing or shunting) tonic GABA conductance, which is normalized by 100 nM ALLO, and is capable to antagonize the tonic NMDA-R-mediated excitation of these cells.

The molecular pathways involved in the dynamic plasticity of δ-GABA_A_Rs remain unknown and will constitute the subject of future investigations. Fast modifications in the endocytosis machinery may play a role in the initial δ-GABA_A_Rs downregulation (Gonzalez et al., 2012). Interestingly it seems that long-term exposure to NMDA induces δ-GABA_A_R mRNA expression in cultured neurons (Gault and Siegel, 1997). A similar mechanism may play a role in postpartum upregulation of δ-GABA_A_Rs to virgin levels in INs.

Changes in γ oscillations have been reported in various neurological and psychiatric disorders (Uhlhaas and Singer, 2010, 2012), and range from poor mnemonic performance to psychosis and schizophrenia. Here we show how diminished δ-GABA_A_Rs and increased NS levels are balanced during pregnancy and postpartum so that the tonic inhibition of PV+BCs and ultimately γ oscillation frequency are kept constant. Several symptoms typical of pregnancy and postpartum pathology may be ascribed to altered γ oscillations. If postpartum depression is a condition resulting from a mismatch between rapidly plummeting NS levels and the need to restore the number of δ-GABA_A_Rs to pre-pregnancy levels (Maguire and Mody, 2008), then the plasticity of IN δ-GABA_A_Rs may also follow a similar course in such pathological conditions. Accordingly, in schizophrenic patients the abnormal γ activity and the high occurrence of depressive behaviors may be a sign of comorbidity between these two conditions (Buckley et al., 2009). As cortical γ activity can be easily recorded with scalp EEG, changes in these oscillations in subjects predisposed to postpartum depression and epilepsy may help identify patients at risk, and could serve to devise δ-GABA_A_R-specific pharmacological strategies for treating some of the major symptoms of the disease.

## Concluding remarks

We have demonstrated a homeostatic down-regulation of δ-GABA_A_Rs in PV+ INs in late pregnancy. We thus established that these cells control the surface expression of δ-GABA_A_Rs without expressing the highly plastic α4 subunit partner of δ-GABA_A_Rs. We provide evidence that γ oscillation frequency recorded *in vitro* is artificially increased in slices of pregnant animals because of the acute withdrawal from plasma precursors. Adding back the levels of NS found in pregnancy normalizes γ frequency, showing the finely balanced homeostatic reduction in δ-GABA_A_Rs expression. Giving the large amount of evidence linking altered γ oscillations with dysfunctional network processing, our findings have the potential to define neurological performance and precipitate preexisting neurological and psychiatric conditions during and after pregnancy. Milder and shorter NS fluctuations such as those typical of the ovarian cycle and stress could also modify δ-GABA_A_Rs expression on PV+ INs and consequently influence network oscillatory behavior, depending on the accuracy of time coupling of NS to δ-GABA_A_Rs expression. The δ-GABA_A_Rs plasticity on PV+ INs may not be restricted to hippocampal area CA3, making it highly probable that similar modulations of γ oscillations take place on a wider scale. Recent discovery of differential pharmacology between α4/δ-GABA_A_Rs and α 1/δ-GABA_A_Rs (Jensen et al., 2013) may help elucidate of their role in the control of emergent properties of neuronal networks in brain areas where both receptor combinations are present.

## Author contributions

Isabella Ferando and Istvan Mody designed research, Isabella Ferando performed experiments, Isabella Ferando and Istvan Mody analyzed data, Isabella Ferando and Istvan Mody wrote the paper.

### Conflict of interest statement

The authors declare that the research was conducted in the absence of any commercial or financial relationships that could be construed as a potential conflict of interest.
